# The uniquely predictive power of evolutionary approaches to mind and behavior

**DOI:** 10.3389/fpsyg.2014.01372

**Published:** 2014-11-28

**Authors:** Ian D. Stephen, Mehmet K. Mahmut, Trevor I. Case, Julie Fitness, Richard J. Stevenson

**Affiliations:** Department of Psychology, Macquarie UniversitySydney, NSW, Australia

**Keywords:** evolutionary psychology, e-cognition, ethology, explanatory power, proximate/ultimate

## Introduction

Barrett et al. (2014) argue that the primary contribution of evolutionary psychology (EP), as defined by the Santa Barbara school (Cosmides and Tooby, 1987; see also Laland and Brown, 2011) is the conception of the mind as a collection of separate, domain-specific mental modules that evolved to solve specific adaptive problems. This, they argue, means that EP does not represent a true alternative to computational models of mind and is therefore not a significant advance on more traditional cognitive approaches. Instead, they recommend that e-cognition, and in particular the concept of the extended mind, can best enhance our understanding of human mind and behavior. While we appreciate Barrett et al.'s enthusiasm for an interesting and relatively new approach to understanding mind and behavior, we argue here that, independent of the veracity of the concept of massive modularity (which is an empirical question; Barrett et al., 2014; Burke, 2014; Stephen, 2014), an evolutionary approach provides a substantial advance in the understanding of mind and behavior. Here, we make two main arguments. First, we argue that a full understanding of mind, brain and behavior requires the consideration of all four of Tinbergen's levels of explanation, which can only be achieved by approaching the problem through the lens of evolution (independent of the assumption of massive, domain-specific modularity, or of any other model). Second, we argue that the embodied cognition approach advocated by Barrett et al. (2014) is actually better understood as an extension of traditional *causal* (mechanistic), and *ontogenetic* (developmental) approaches than as a revolutionary approach in its own right, and therefore is best examined through the lens of evolution.

## The value of evolutionary approaches to mind and behavior

In what is now widely considered the foundational document of human ethology, Niko Tinbergen makes the case that behavior can be addressed at four different explanatory levels (Tinbergen, 1963). In addition to the *causal* (or mechanistic) and *ontogenetic* (developmental) levels of explanation that are typical of modern psychology, Tinbergen proposed that a full understanding of behavior requires that we consider two additional, evolutionary levels of explanation. The *phylogenetic* level considers the evolutionary history of the behavior, and the *functional* level considers what he calls the survival value, or what modern evolutionists would call the fitness value or selective value of the behavior (though more recently, O'Brien and Gallup, 2011, have suggested that the role of culture represents a fifth level of explanation). While Barrett et al. (2014) assert that the primary advance offered by EP is the conception of the mind as massively modular, we suggest that the defining feature of evolutionary approaches to psychology is simply the application of the evolutionary concepts of selection and fitness to human behavior. This approach allows us to address human psychology through Tinbergen's *phylogenetic* and *functional* levels of explanation, providing novel hypotheses and a more thorough understanding of the subject. Despite rarely being acknowledged directly, these principles are applied in a range of evolutionary approaches to mind and behavior (e.g., Stephen, 2013).

This application of evolutionary concepts to psychology is not reliant on the assumption of massive, domain-specific modularity, since predictions derived from such an assumption are often identical to those derived from evolutionary approaches based on plasticity, domain-generality, and cultural evolution. What changes is merely the level on which selection is assumed to act. Whereas a Santa-Barbara school Evolutionary Psychologist would think of selection as acting upon genes coding for domain-specific, yet flexible, mental modules, a more domain-general evolutionary approach would see selection as acting upon the behaviors themselves. In either case, the behaviors and cognitions selected for and against remain the same (Burke, 2014; Stephen, 2014). Indeed, the majority of research in this area does not make direct assumptions about massive modularity or lack thereof (Burke, 2014; Stephen, 2014). The question of whether the mind is massively modular and domain-specific or plastic and culturally selected remains, then, an important empirical question (Barrett et al., 2014), but one that is tangential to the issue of whether evolution offers a useful contribution to the study of mind and behavior (Stephen, 2014).

Irrespective, then, of the unit of selection, we suggest that an evolutionary approach can offer unique insights into understanding and predicting behavior. Indeed, most of the added value brought by an evolutionary approach is reflected in the two neglected aspects (for psychology at least) of Tinbergen's ethological approach to behavior. Evolutionary psychologists are perhaps with good reason shy of admitting that consideration of function may be useful when thinking about behavior. Much of this concern relates to *a posteriori* reasoning, and the criticism of “just so stories.” However, a consideration of function *a priori* can be a powerful aid to theorizing and hypothesis generation. In research on disgust, for example, the principal driver behind studying this emotion's relationship with the immune system was based upon the idea that disgust *functions* to aid disease avoidance (Stevenson et al., 2011). Without a consideration of the functional value of this emotion, such avenues of enquiry would not have been envisaged.

A further benefit of an evolutionary approach is in consideration of the phylogenetic origin of a particular behavior. This seems to be a more neglected line of reasoning within human EP, but it can be highly instructive. Again, take disgust as an illustrative example. It has been argued that disgust is a uniquely human emotion, with a small phylogenetic “tail” (Rozin et al., 2010). This “tail” extends into other mammals (and beyond) and has been termed “distaste.” Distaste functions primarily as a specific defense against consuming bitter (poisonous) food. However, mammals and indeed all animals face similar pathogen threats to humans, and it would be surprising if we did not also share some of the same basic behaviors to avoid getting sick. In fact, a very extensive set of disease avoidant behaviors have been documented in animals (e.g., Hart, 2011) but surprisingly, almost no research has explored whether the emotion of disgust plays a role in animal disease avoidance. Not only, then, can the idea of phylogenetic continuity act to stimulate new avenues for research, it can also act to complement the functional approach. For example, if animals do use disgust to assist disease avoidance, this would be consistent with the functional interpretation of disgust in humans. Further, Schaller and Murray's (2008) finding of regional personality differences corresponding to pathogen prevalence offers a clear illustration of the use of evolutionary theorizing to generate novel predictions across multiple levels to draw a connection between traditionally disparate domains. Crucially, *none* of this theorizing relies upon a commitment to any particular theory of the unit of selection.

Intra-species color cues may be taken as another example of a *phylogenetic* approach that has advanced our understanding of human behavior. Color is frequently used to convey information in non-human animals. For example, male hooded vultures have highly vascularized, exposed skin on their heads, which flush red during antagonistic encounters, and male ostriches show redder necks during the mating season, suggesting that this hemoglobin-based coloration is a cue to dominance and fertility (Negro et al., 2006). A *phylogenetic* approach allows us to make predictions about the kinds of perceptual biases and behaviors that we expect to see in humans and other species. We know that the majority of mammals have only dichromatic vision that precludes the differentiation of red from green (Carroll et al., 2001), whereas old world, and some new world, primates have trichromatic vision. The *phylogenetic* approach thus allows us to predict that we may see red cues in primates, including humans and old world monkeys, but not in non-primate mammals, and new world monkeys with dichromatic vision (Changizi et al., 2006). This is indeed what we see. The red coloration of mandrills' faces increases with higher position in the dominance hierarchy and with higher testosterone (Setchell and Dixson, 2001). During antagonistic confrontations, the less red male is more likely to back down (Setchell and Wickings, 2005), and female mandrills prefer to mate with redder faced males, irrespective of alpha status (Setchell, 2005). Similarly, in humans, we see redder facial skin in men interpreted as appearing more aggressive, dominant, attractive (Stephen et al., 2012), and healthy (Stephen et al., 2009a,b). Indeed, it has been suggested that one evolved function of trichromatic vision in primates may be to enable individuals to identify color-based social cues (Changizi et al., 2006).

This prediction of human psychological traits based on *phylogenetic* approaches, then, allows enhanced predictive power and greater understanding of the psychology of humans.

## e-cognition's proximal explanatory nature

Barrett et al. (2014) suggest that an alternative to the standard computational theories of mind (in which they include Santa Barbara school EP) is the various e-cognition approaches. They focus on one form, the extended mind hypothesis (e.g., Clark and Chalmers, 1998), which holds that the boundaries of cognition extend well beyond the central nervous system, so that the body and the environment form a coupled system that governs behavior. The main benefit of such an approach, according to Barrett et al. (2014), is that it encompasses the complex array of external features (e.g., written language, visual aids, etc.) that shape human behavior in the current environment.

While we agree that e-cognition approaches offer potentially interesting ways of understanding behavior, we would also argue that they are essentially elaborations of the computational models of mind that Barrett et al. (2014) criticize, representing extensions of Tinbergen's (1963) *causal* (mechanistic), and *ontogenetic* (developmental) levels of explanation. Extending the boundary of cognition to include objects that are not typically considered as part of the cognitive system (e.g., a shopping-list memory aid) does not address a *functional* or *phylogenetic* level of analysis, any more than does a standard computational approach. This can only be achieved by studying behavior through the evolutionary concepts of selection and fitness. As such, Barrett et al.'s suggested alternative to EP—e-cognition—does not represent a true alternative to computational models of mind, but rather an extension of these approaches that should be best approached through the lens of evolutionary theory. In this way, Barrett et al.'s (2014) conception of e-cognition as an alternative to evolutionary approaches to cognition and behavior mischaracterizes e-cognition as an ultimate explanatory framework, when it should properly be considered proximal (see Scott-Phillips et al., 2011, for similar arguments in response to previously proposed alternative ultimate explanatory frameworks, such as cultural evolution and epigenetics).

## Conclusions

Accordingly, we argue that evolutionary approaches provide significant additional predictive and explanatory value above standard computational models by allowing researchers to address the *phylogenetic* and *functional* levels of explanation. Evolutionary approaches to mind and behavior, then, go well beyond existing approaches in their potential to provide an understanding, not necessarily of the how, but of the why, humans behave as they do in an unpredictable world. Consider, for example, the richness and complexity of human emotion: forged over the course of human evolution and responding to present day triggers, the passions drive behavior—albeit often to dysfunctional ends within modern societies (e.g., Fitness and Case, 2003). Understanding such diverse emotions as anger, jealousy, hate, love, disgust, or shame as evolution's executioners (Wright, 1995) provides us with an answer to the question of the “why” of behavior that cannot be addressed by only *causal* and *ontogenetic* levels of analysis. In short, e-cognition accounts, along with other approaches that do not hold the evolutionary principles of selection and fitness as central represent only extensions of the more proximate explanations of mind and behavior, rather than providing the fuller understanding of cognition and behavior that ensues from *phylogenetic* and *functional* level of explanations. Further, one extraordinary achievement of evolutionary approaches to mind and behavior has been to demonstrate the commonalities shared by human beings across time and space as a function of the adaptive problems they have always faced, and continue to face, as social animals who depend upon one another for their survival. Certainly, humans today are confronted with a material, technological world that could not be imagined by humans who lived thousands of years ago. However, a baby from our recent evolutionary past miraculously transported through time to a modern Western environment would still crave attachment and belonging, experience, and respond to the world and others through her senses and feelings, and learn through language how to interpret, communicate, and function more or less adaptively in that environment, just as babies raised in regions geographically distant from their ancestral homelands do today. No doubt she would also help her parents program their latest iPhone along the way.

### Conflict of interest statement

The authors declare that the research was conducted in the absence of any commercial or financial relationships that could be construed as a potential conflict of interest.
